# Nutritional Management for Pediatric Biliary Atresia Patients Preparing for Liver Transplantation

**DOI:** 10.3390/children12030391

**Published:** 2025-03-20

**Authors:** Uyory Choe

**Affiliations:** Department of Food and Nutrition, College of Biomedical and Health Science, Konkuk University, Chungju 27478, Republic of Korea; foodtech@kku.ac.kr; Tel.: +82-(43)-840-3581

**Keywords:** biliary atresia, liver transplantation, nutritional deficiencies, medium-chain triglycerides (MCT), fat-soluble vitamins

## Abstract

Biliary atresia, a rare pediatric liver condition, results in blocked bile ducts, impeding bile secretion and causing significant nutritional challenges. This perspective emphasizes the critical role of nutrition in supporting children with biliary atresia awaiting liver transplantation. The liver’s multifaceted functions in energy metabolism, vitamin storage, and waste excretion emphasize the importance of tailored dietary interventions. Medium-chain triglyceride (MCT) oil serves as a crucial energy source, addressing fat malabsorption, while specialized water-soluble formulations deliver essential fat-soluble vitamins. Additionally, weaning strategies and developmental food practices are discussed to ensure optimal growth and development despite dietary restrictions. Feeding assistance through nasogastric or gastrostomy tubes is explored as a means to combat malnutrition and support liver function. The collective efforts of caregivers and healthcare providers are pivotal in preparing these children for successful liver transplantation, aiming to secure their future health and quality of life.

## 1. Introduction

Biliary atresia is a rare disease affecting infants, characterized by blocked or absent bile ducts. It typically occurs between 4 and 8 weeks after birth. Statistically, it is more common in females than males and has a higher incidence among Asian patients compared to other ethnic groups [1].

The bile ducts transport bile, a digestive fluid produced by the liver, to the duodenum. Bile is essential for nutrient absorption and contributes to detoxification by excreting cholesterol, fat-soluble toxins, and heavy metals [2]. Therefore, biliary atresia limits bile and toxin excretion from the liver, causing liver damage.

Initial symptoms of biliary atresia include jaundice and changes in stool and urine color. Jaundice results from the accumulation of bilirubin, derived from hemoglobin, in the body due to impaired bile excretion. Stools appear pale white or gray because of the lack of bilirubin, while urine becomes dark yellow or brown as more bilirubin is excreted than normal people through it [1].

Kasai surgery, developed by Japanese surgeon Morio Kasai in the 20th century, is a treatment for biliary atresia. This surgery involves removing the obstructed bile ducts and connecting the liver directly to the intestine to enable bile drainage. It is crucial for parents to recognize the symptoms of biliary atresia early and ensure their child undergoes Kasai surgery promptly [3]. Delays in surgery can lead to progressive liver damage, reducing its effectiveness. Furthermore, Kasai surgery is not optional but necessary—without it, most children with biliary atresia do not survive beyond two years.

The prognosis after Kasai surgery is often categorized using the “one-third rule” [4]: First third: Bile drainage is successful, liver function remains normal, and the patient survives without needing a liver transplant. Second third: Bile drainage is initially successful, and liver function remains normal for a time, but fibrosis progresses over the years, requiring a liver transplant in childhood or adolescence. Last third: Bile drainage is minimal even after Kasai surgery, necessitating a liver transplant within the first year of life.

Throughout life, humans require consistent nutrient intake, especially during childhood, when diverse nutrients are essential for growth and development. However, children in the last one-third category of the “one-third rule” often experience significant nutritional deficiencies due to inadequate bile excretion even after Kasai surgery. Additionally, liver transplants are not feasible for very small children, and the success rate is higher when the child has achieved adequate growth. Therefore, from the time of Kasai surgery at 2–3 months of age until about one year, efforts must focus on minimizing nutritional deficiencies to support normal growth and development.

The purpose of this perspective is to provide healthcare providers and families of children awaiting liver transplantation due to biliary atresia with information on the liver’s nutritional functions, supportive foods, and essential nutrients. This piece of information will help affected children achieve optimal growth and development so that the child can receive a successful liver transplant and return to normal life.

## 2. Functions of the Liver

**Figure 1:**
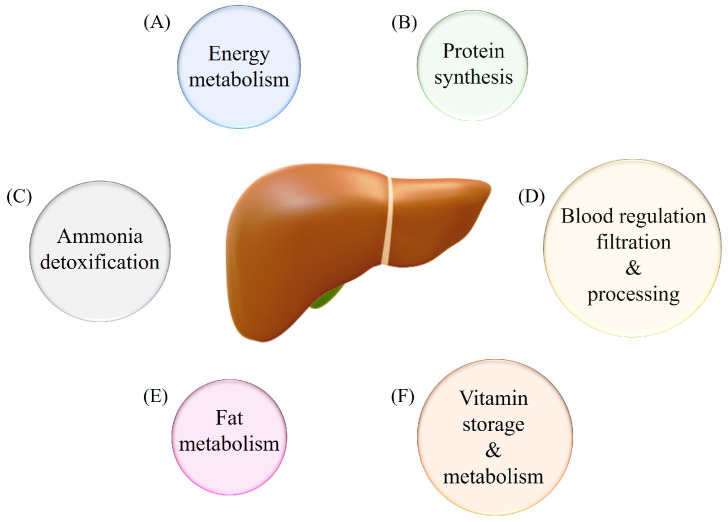
Functions of the liver. The liver’s functions are illustrated in circles and those include (A) energy metabolism, (B) protein synthesis, (C) ammonia detoxification, (D) blood regulation, filtration, and processing, (E) fat metabolism, and (F) vitamin storage and metabolism.

The liver is an important organ that governs many physiological functions. These include energy metabolism, protein synthesis, ammonia detoxification, blood regulation, filtration, and processing, as well as fat metabolism (Figure 1). In this section, the functions of the liver are discussed.

### 2.1. Energy Metabolism

Various forms of carbohydrates in food, especially starches, are broken down into monosaccharides such as glucose and fructose by carbohydrate-degrading enzymes like amylase, which are secreted in the salivary glands and small intestine [5]. These monosaccharides are absorbed through the small intestine and transported to the liver via the bloodstream. Some glucose is absorbed into liver cells via the GLUT2 transporter. The absorbed glucose is either used immediately for energy or converted into glycogen for storage in the liver. When blood sugar levels drop, the stored glycogen is broken down back into glucose to restore blood sugar levels.

Additionally, the liver can synthesize glucose from non-carbohydrate substances (e.g., lactate, amino acids, glycerol) through a process called gluconeogenesis [6]. For instance, lactate is transported to the liver through the bloodstream, converted into pyruvate with the help of lactate dehydrogenase, and then into glucose. Similarly, amino acids from muscle protein breakdown are transported to the liver and contribute to glucose production through deamination or transamination.

### 2.2. Protein Synthesis

The liver also plays a key role in protein synthesis. It produces various proteins, including plasma proteins, blood clotting proteins, transport proteins, immune proteins, acute-phase proteins, and metabolic regulatory proteins [7]. For example, albumin, the most abundant plasma protein, helps transport fatty acids, steroids, and bilirubin in the bloodstream while maintaining osmotic pressure. Fibrinogen, a clotting protein, is converted into fibrin by thrombin to form blood clots. Lastly, C-reactive protein (CRP), synthesized by the liver, is an important immune protein used as a marker in blood tests to evaluate inflammation and infection.

### 2.3. Ammonia Detoxification

Ammonia is generated during protein metabolism when the amino group of amino acids is removed. It can also be produced by bacterial activity in the gut, the breakdown of nitrogen compounds, or nucleic acids [8]. As ammonia is toxic, the liver converts it into urea, a non-toxic compound excreted in urine by the kidneys.

### 2.4. Blood Regulation, Filtration, and Processing

The liver holds about 10–15% of the body’s total blood volume. In situations like exercise, bleeding, or low blood pressure, the liver releases stored blood to supplement the body’s needs. It also filters and processes various substances carried in the blood [9]. For instance, nutrients absorbed from food and toxins introduced externally are transported to the liver through the portal vein. Also, the hepatic artery supplies oxygen-rich blood to the liver. Finally, the hepatic vein carries filtered blood from the liver back to the heart.

### 2.5. Fat Metabolism

When carbohydrates are consumed in excess, the liver stimulates fatty acid synthesis through insulin secretion. These carbohydrates are converted into triglycerides and cholesterol for storage or transport. Conversely, during energy shortages, glucagon triggers the breakdown of stored fats for energy use [10].

### 2.6. Vitamin Storage and Metabolism

In addition to energy metabolism, the liver stores and regulates fat-soluble vitamins (A, D, E, K) [11]. These vitamins are absorbed in the small intestine after emulsification by bile and transported to the liver via the lymphatic and circulatory systems. The liver stores these vitamins as needed and distributes them to tissues. The functions and food sources of fat-soluble vitamins are shown in Table 1.

### 2.7. Fat-Soluble Vitamins

Vitamin A: Exists as carotenoids and retinoids, which are fat-soluble compounds. Vitamin A contributes to eye protection, epithelial cell maintenance, cell division, and growth promotion [12]. Deficiency can cause night blindness, dry eyes, keratinized skin, and growth delays in infants. Rich sources include carrots, seaweed, and butter.

Vitamin D: Known as calciferol, it regulates calcium and phosphorus balance and is essential for bone formation. Deficiency can lead to rickets, osteomalacia, osteoporosis, and growth issues in children [13]. Sources include mushrooms, yeast, milk, eggs, butter, and fortified products.

Vitamin E: Known for its antioxidant properties, it prevents oxidation of unsaturated fatty acids, phospholipids, and vitamin A. It also helps prevent aging and aids iron absorption. Deficiency may cause infertility, muscle atrophy, and anemia [14]. Sources include wheat germ, vegetable oils, margarine, and shortening.

Vitamin K: Discovered in 1935, it plays a role in blood clotting. Types include K1 (phylloquinone) from plants, K2 (menaquinone) from animals or intestinal bacteria, and synthetic K3–K7. Deficiency results in delayed blood clotting [15]. Sources include green leafy vegetables, seaweed, eggs, and meat.

### 2.8. Malnutrition of Biliary Atresia Patients

The liver produces bile, which is stored in the gallbladder after being transported through the bile ducts. During food consumption, bile is released to emulsify triglycerides with water, allowing pancreatic lipase to break down the emulsified triglycerides into fatty acids and glycerol. These are then encapsulated by bile salts to form micelles, which are transported to the intestinal epithelial cells for absorption. However, patients with biliary atresia experience difficulty in bile secretion, leading to impaired emulsification and subsequent absorption of triglycerides and water [16].

Beyond nutrient absorption, bile also plays a crucial role in waste excretion. Bilirubin, a byproduct of red blood cell breakdown, is excreted with bile and contributes to stool coloration. Excess cholesterol is either excreted in bile or converted into bile acids, and detoxified substances are eliminated through bile [17]. When the bile ducts are obstructed, patients face not only nutritional challenges but also issues with waste excretion. Even after undergoing Kasai surgery, if bile secretion remains insufficient, these problems can worsen over time, making it difficult to maintain the health needed for liver transplantation. Thus, if the post-surgery prognosis is poor, early intervention focusing on nutritional support and development is critical to prolong the time until a transplant is possible.

Food is essential for life, providing both nutrition and enjoyment, particularly for children, for whom food can also be a form of play. From birth to one year of age, food is crucial not only nutritionally but also developmentally [18]. Developmentally, exposure to various ingredients allows infants to explore and learn about different colors, tastes, aromas, and textures, helping them establish preferences. Most infants consume breast milk or formula until about six months of age, transition to weaning foods between 6 and 8 months, start trying diverse foods such as puffs and freeze-dried yogurt between 8 and 10 months, and begin eating solid foods by 10–12 months. By 12 months, most children can eat a wide range of foods [19]. However, adequate nutrient absorption is a prerequisite for these developmental processes. Without proper nutrient absorption, malnutrition and developmental delays can occur.

Fat is the primary calorie source for infants up to one year old, comprising 50% of the calories in breast milk and formula [19]. Fat is vital for brain development, nervous system formation, and hormone synthesis [20]. However, fat absorption requires bile emulsification and biliary atresia patients struggle with fat absorption due to insufficient bile secretion. To address this, the European Society for Paediatric Gastroenterology, Hepatology and Nutrition (ESPGHAN) and the European Society for Clinical Nutrition and Metabolism (ESPEN) suggest carefully adjusted nutritional supplementation for pediatric patients with biliary atresia before undergoing liver transplantation [21]. Among these recommendations, the ESPGHAN guidelines emphasize ensuring adequate caloric intake through formulas enriched with medium-chain triglycerides (MCT), considering the reduced absorption efficiency of long-chain fatty acids [22]. The energy requirement for healthy infants is approximately 100–120 kcal/kg/day, whereas pediatric patients with biliary atresia are recommended to consume at least 130–150 kcal/kg/day. When calculated in percentage terms, this means they need to consume approximately 30–50% more calories than healthy infants [23,24].

Moreover, among fatty acids, omega-3 (n-3) and omega-6 (n-6) fatty acids play a critical role in pediatric growth and development as they belong to essential fatty acids (EFAs). These essential fatty acids can be supplemented by increasing intake through emulsified or water-soluble formulations, as well as by using specialized infant formulas enriched with omega-3 and omega-6.

Additionally, ESPGHAN and ESPEN highlight the importance of supplementing fat-soluble vitamins (A, D, E, K) for biliary atresia patients, as conventional forms may have low absorption rates. Therefore, they recommend using water-soluble or specially formulated alternatives to enhance absorption. The recommended dosage should be tailored individually based on the patient’s nutritional status and regular monitoring of vitamin levels.

### 2.9. Role of Medium Chain Triglycerides (MCT) Oil in Nutrition

Triglycerides are composed of one glycerol molecule and three fatty acids. The fatty acids in most plant- and animal-based oils are long-chain fatty acids with carbon chains of 14–18 atoms, but a larger number is possible [25]. In contrast, MCT oil, commonly derived from palm or coconut oil, contains medium-chain fatty acids with carbon chains of 6–12 atoms, making digestion and absorption easier. Unlike long-chain fatty acids, which require bile emulsification for digestion, MCT oil is more accessible to lipase enzymes, facilitating hydrolysis. Furthermore, medium-chain fatty acids bypass the lymphatic system and are directly transported to the liver via the portal vein [26]. For biliary atresia patients awaiting liver transplants, MCT oil provides sufficient calories to support normal development.

MCT oil is included in many specialized infant formulas available in the market. If such formulas are unavailable, refined MCT oil can be added to standard formulas. However, MCT oil’s slippery texture and strong taste may reduce its palatability, leading to rejection by infants. Thus, in the future, improving the taste and acceptance of MCT oil-based products is needed for the support of biliary atresia patients.

### 2.10. Challenges with Fat-Soluble Vitamins

In addition to fat absorption issues, biliary atresia patients face significant challenges with fat-soluble vitamins (A, D, E, K), which require bile for absorption. Vitamins A and D are primarily stored in the liver, and cirrhosis exacerbates deficiencies of these vitamins [27]. Unlike medium-chain triglycerides, there are no alternate pathways for absorbing fat-soluble vitamins, making their deficiencies more severe. Advances in pharmaceutical technology have enabled the development of water-soluble forms of fat-soluble vitamins to improve absorption, but these remain limited in availability [28].

High concentrations of fat-soluble vitamin supplements are often required for biliary atresia patients, but their taste can be unpleasant for infants. These supplements are often formulated with fruit flavors to mask their bitterness, but the strong flavors may be overwhelming for young infants. Additionally, the high concentrations required can cause vomiting if mixed with formula, leading to further nutritional loss. It is crucial for caregivers, nutritionists, and healthcare providers to identify and adjust the dosage of these vitamins based on the infant’s tolerance and ability to absorb nutrients effectively.

### 2.11. Improving the Digestibility of Fat and Fat-Soluble Vitamins via Ursodeoxycholic Acid

Ursodeoxycholic acid (UDCA), also known as ursodiol, can be used as a bile acid replacement therapy to aid in fat and fat-soluble vitamin digestion and provide hepatoprotective effects in pediatric patients with biliary atresia [4]. UDCA is a hydrophilic bile acid that helps alleviate cholestasis commonly observed in biliary atresia patients and improves liver function by reducing hepatocellular stress through its antioxidant and anti-inflammatory properties. However, the efficacy of UDCA varies among individuals. Thus, its therapeutic application should be determined based on the patient’s specific condition.

### 2.12. Weaning Foods and Solid Foods

At six months of age, infants typically begin transitioning to weaning foods. Weaning foods supplement the nutrients from breast milk or formula, provide additional calories, and support growth and development. Introducing small amounts of solid food also helps infants develop chewing and swallowing skills, strengthening oral muscles [29]. During this stage, infants start forming eating habits, interacting with family during meals, and exploring textures and flavors through play. This developmental stage is critical for lifelong eating behaviors [30]. Currently, there are numerous baby food products from different companies. Easily accessible puree flavors include banana, sweet potato, apple, butternut squash, green bean, carrot, peach, mango, prune, and others. In addition to these original flavors, there are mixed ones as well. Around 8 months, babies can start snacks such as puffs and freeze-dried yogurt. These products are not only good for normal development for the baby, but also excellent sources of vitamins and minerals as these products are rich in them.

By 10 months of age, infants in good health can begin eating solid foods, such as steamed carrots, broccoli, potatoes, chopped bananas, apples, avocados, and cheese [31]. However, dietary restrictions may still apply to biliary atresia patients with severe liver dysfunction. When bile excretion is significantly impaired, bilirubin, lipid byproducts, and toxins accumulate in the liver, causing inflammation and liver damage. This progression to cirrhosis further limits dietary options.

Therefore, for biliary atresia patients, caregivers must balance the child’s medical needs with normal developmental experiences. Despite the challenges of their condition, providing varied weaning foods, snacks, as well as solid foods, and engaging in mealtime interactions can foster normal growth and happy moments for both the child and the family.

### 2.13. Use of Nasogastric (NG) and Gastrostomy (G) Tubes in Biliary Atresia Patients

For biliary atresia patients, Kasai surgery may temporarily improve bile secretion, but cirrhosis often develops, leading to poor digestion and appetite loss. These issues can result in malnutrition and hinder growth and development. In such cases, feeding tubes like nasogastric (NG) tubes or gastrostomy (G) tubes may be necessary [32].

NG tube: Inserted through the nose into the stomach, NG tubes are easy to insert and remove, making them suitable for home use. However, they may cause discomfort and are prone to accidental removal by the infant.

G tube: Surgically inserted directly into the stomach through the abdominal wall, G tubes offer a more comfortable long-term solution but require surgery and carry a risk of infection.

Both options facilitate the administration of essential nutrients, medications, and fat-soluble vitamins, but careful monitoring of feeding volume and speed is critical to avoid complications like vomiting, which could exacerbate malnutrition [33].

### 2.14. Enterohepatic Axis Dysfunction and the Role of Prebiotics and Probiotics

Enterohepatic axis dysfunction is a common issue in pediatric patients with biliary atresia, characterized by reduced bile acid levels in the intestine, leading to an imbalance in the gut microbiota. This microbial dysbiosis can promote bacterial overgrowth, which in turn impairs nutrient absorption and triggers persistent inflammatory responses. Studies have shown that an increase in pathogenic bacteria in the gut is associated with a higher risk of systemic infections (sepsis), cholestatic hepatitis, and post-liver transplantation complications [16]. Therefore, strategies to regulate gut microbiota and suppress inflammation are essential.

One strategy involves a dietary intervention to restore microbial balance. The consumption of prebiotics, which supports the growth of beneficial gut bacteria, can help improve gut health, while probiotics containing beneficial bacteria such as *Lactobacillus* and *Bifidobacterium* can prevent the proliferation of harmful pathogens and enhance immune function [21]. Another approach is antibiotic therapy. If dietary interventions are insufficient and persistent intestinal infections or bacterial overgrowth are suspected, localized antibiotics such as rifaximin can be used to control pathogenic bacteria in the gut [17]. These approaches help reduce the risk of infections associated with enterohepatic axis dysfunction and contribute to optimizing the nutritional status of pediatric patients with biliary atresia by improving gut microbial balance.

## 3. Conclusions

Maintaining the health of biliary atresia patients throughout the journey from Kasai surgery to liver transplantation is critical. This requires the combined efforts and dedication of caregivers and healthcare providers. Identifying the necessary nutrients and addressing deficiencies with tools like MCT oil and water-soluble forms of modified fat-soluble vitamins can minimize malnutrition. Additionally, incorporating varied food experiences can promote both physical growth and developmental milestones, creating an optimal environment for transplantation.

Beyond nutritional interventions, maintaining gut health is essential in optimizing outcomes for biliary atresia patients. Enterohepatic axis dysfunction frequently leads to gut dysbiosis, increasing the risk of systemic infections and nutrient malabsorption. Strategies such as probiotic and prebiotic supplementation, along with targeted antibiotic therapy when necessary, can help regulate gut microbiota and mitigate inflammation. Furthermore, bile acid supplementation with ursodeoxycholic acid (UDCA) has shown potential benefits in enhancing fat digestion and protecting hepatic function, although its application should be tailored to individual patient needs.

While this journey demands relentless effort and commitment from families and caregivers, the goal is to ensure biliary atresia children get enough nutrition and provide a brighter future. A multidisciplinary approach, including pediatric gastroenterologists, dietitians, and transplant specialists, is essential to developing personalized nutritional strategies that enhance survival rates and long-term quality of life. Ongoing research and advancements in nutritional therapy will further refine treatment approaches, offering hope for improved outcomes for these vulnerable patients.

## Figures and Tables

**Table 1:** Types of fat-soluble vitamins, functions, and food sources.

Vitamin	Functions	Food Sources
Vitamin A	Eye protection, epithelial cell maintenance, cell division, growth promotion	Carrots, sweet potatoes, pumpkins, butternut squash, spinach, kale, mangoes, apricots, cantaloupes, papayas, seaweed, eggs, butter
Vitamin D	Regulates calcium and phosphorus balance, essential for bone formation	Mushrooms, yeast, milk, eggs, butter, salmon, tuna, fortified products
Vitamin E	Antioxidant properties, prevent oxidation of unsaturated fatty acids and vitamin A, aid iron absorption	Wheat germ, vegetable oils, margarine, shortening, almonds, sunflower seeds, hazelnuts, peanuts, broccoli, spinach, asparagus, kale, avocados, kiwis
Vitamin K	Blood clotting	Green leafy vegetables, seaweed, eggs, meat, cheese

## Abbreviations

The following abbreviations are used in this manuscript:

EFAs	Essential Fatty Acids
G	Gastrostomy
MCT	Medium Chain Triglycerides
NG	Nasogastric
UDCA	Ursodeoxycholic Acid
